# The Instability of Dimeric Fc-Fusions Expressed in Plants Can Be Solved by Monomeric Fc Technology

**DOI:** 10.3389/fpls.2021.671728

**Published:** 2021-07-09

**Authors:** Pia Gattinger, Shiva Izadi, Clemens Grünwald-Gruber, Somanath Kallolimath, Alexandra Castilho

**Affiliations:** ^1^Department of Applied Genetics and Cell Biology, Institute for Plant Biotechnology and Cell Biology, University of Natural Resources and Life Sciences Vienna, Vienna, Austria; ^2^Division of Immunopathology, Department of Pathophysiology and Allergy Research, Center for Pathophysiology, Infectiology and Immunology, Medical University of Vienna, Vienna, Austria; ^3^Department of Plant Genetics and Breeding, Faculty of Agriculture, Tarbiat Modares University, Tehran, Iran; ^4^Division of Biochemistry, Department of Chemistry, University of Natural Resources and Life Sciences Vienna, Vienna, Austria

**Keywords:** *Nicotiana benthamiana*, Fc-fusion proteins, EPO-Fc, protein instability, protein linkers

## Abstract

The potential therapeutic value of many proteins is ultimately limited by their rapid *in vivo* clearance. One strategy to limit clearance by metabolism and excretion, and improving the stability of therapeutic proteins, is their fusion to the immunoglobulin fragment crystallizable region (Fc). The Fc region plays multiple roles in (i) dimerization for the formation of “Y”-shaped structure of Ig, (ii) Fc-mediated effector functions, (iii) extension of serum half-life, and (iv) a cost-effective purification tag. Plants and in particular *Nicotiana benthamiana* have proven to be suitable expression platforms for several recombinant therapeutic proteins. Despite the enormous success of their use for the production of full-length monoclonal antibodies, the expression of Fc-fused therapeutic proteins in plants has shown limitations. Many Fc-fusion proteins expressed in plants show different degrees of instability resulting in high amounts of Fc-derived degradation products. To address this issue, we used erythropoietin (EPO) as a reporter protein and evaluated the efforts to enhance the expression of full-length EPO-Fc targeted to the apoplast of *N. benthamiana*. Our results show that the instability of the fusion protein is independent from the Fc origin or IgG subclass and from the peptide sequence used to link the two domains. We also show that a similar instability occurs upon the expression of individual heavy chains of monoclonal antibodies and ScFv-Fc that mimic the “Y”-shape of antibodies but lack the light chain. We propose that in this configuration, steric hindrance between the protein domains leads to physical instability. Indeed, mutations of critical residues located on the Fc dimerization interface allowed the expression of fully stable EPO monomeric Fc-fusion proteins. We discuss the limitations of Fc-fusion technology in *N. benthamiana* transient expression systems and suggest strategies to optimize the Fc-based scaffolds on their folding and aggregation resistance in order to improve the stability.

## Introduction

Fc-fusion proteins are bioengineered polypeptides composed of the crystallizable fragment (Fc) region of human immunoglobulin (Ig) G antibody (hinge-CH2-CH3) and another biologically active protein domain, generating a chimeric molecule with unique structure–function properties and therapeutic potential. The amino acid residues in the Fc region and linked protein are very important in the bioactivity and affinity of the fusion proteins. Most frequently, the fused proteins have a therapeutic potential, and attachment to an Fc provides a number of additional biological and pharmacological properties (Dumont et al., 2012; Gan et al., 2015). The Fc-fusion protein technology is used as an important backbone for drug development (Pechtner et al., 2017). Fc-fusions have a prolonged therapeutic activity due to an increased plasma half-life (Yu et al., 2013; Wang et al., 2014; Strohl, 2015) owing to the (i) Fc interaction with the salvage neonatal Fc-receptor [FcRn; (Roopenian and Akilesh, 2007)], (ii) slower renal clearance due to higher molecular weight (Knauf et al., 1988; Kontermann, 2011), and (iii) reduced immunogenicity (Rath et al., 2015). Moreover, the Fc domain folds independently and can improve the solubility and stability of the partner molecule (Zhang et al., 2010). The safety of Fc-fusion proteins in humans has been demonstrated by the development of Fc-based protein drugs (Shapiro et al., 2012; Tuettenberg et al., 2012; Berger et al., 2015). Finally, from a technological perspective, the Fc region allows for easy cost-effective purification by protein-G/A affinity chromatography (Carter, 2011).

Chinese hamster ovary (CHO) cells are the most common host for Fc-fusion protein production, and several therapeutic Fc-fusion proteins on the market are expressed in CHO cells (Walsh, 2018). This is mainly due to the ability of CHO cells to perform complex posttranslational modifications, in particular, protein glycosylation is crucial for the full function of therapeutic products (Sola and Griebenow, 2010). One such example is erythropoietin (EPO), a ∼30-kDa glycoprotein produced primarily by the kidney, which is the principal factor that regulates erythropoiesis by stimulating the proliferation and differentiation of erythroid progenitor cells. Recombinant human EPO-Fc produced in CHO is a dimeric glycosylated polypeptide chain consisting of two mature human EPO molecules linked to the Fc portion of human IgG1 (hinge-CH2-CH3). The fusion protein has a prolonged *in vivo* half-life and enhanced erythropoietic bioactivity in comparison with native human EPO (Salgado et al., 2015).

The market for recombinant therapeutic proteins is increasing rapidly (Moorkens et al., 2017). Plants have proved to be suitable platforms for the production of biopharmaceuticals, and a growing number of plant-made therapeutic proteins have entered clinical trials (Sack et al., 2015; Lomonossoff and D’Aoust, 2016). *Nicotiana benthamiana* is most suited for the rapid large-scale synthesis of recombinant proteins (Bally et al., 2018), and recent advances in plant glycoengineering have allowed the production of plant-derived protein with tailored human-like glycosylation (Montero-Morales and Steinkellner, 2018).

Recombinant expression of EPO-Fc in *N. benthamiana* has been reported previously (Castilho et al., 2011b, 2012, 2013; Nagels et al., 2012). Importantly, glycosylation of plant-derived EPO-Fc has been modulated toward the synthesis of multi-antennary (Castilho et al., 2011b; Nagels et al., 2012), sialylated (Castilho et al., 2013), and mucin-type O-glycosylated (Castilho et al., 2012) glycans. Despite the major achievements on glyco-engineering, attempts to transiently express EPO-Fc in *N. benthamiana* showed that the fusion protein is rather unstable and the intact EPO-Fc fusion is expressed at low levels. Besides EPO-Fc, several other Fc-fusion proteins expressed in plants have shown similar instabilities with varying amounts of free Fc being observed (Lu et al., 2012; De Buck et al., 2013; Kim et al., 2017, 2018; Rattanapisit et al., 2019; Xiong et al., 2019; Diamos et al., 2020; Castilho et al., 2021). These studies have used various therapeutic proteins fused to Fc domains expressed in different ways, differing in the gene construction and transformation method, the nature of the signal peptide, the presence or absence of an endoplasmic reticulum (ER) retention sequence, and the host species. In the present investigation, we examine strategies to stabilize the expression of Fc-fusion proteins in plants using the same therapeutic protein and the same plant expression system. We used EPO as a reporter to evaluate our efforts to enhance the expression of full-length EPO-Fc fusion protein targeted to the apoplast of *N. benthamiana* plants. These included modifications in the hinge region of the IgG1 fusion partner, alternative Fc fusion partners (IgG3 and IgG4), and flexible linkers. Our results highlight that the proteolytic degradation of Fc-fusions most probably arises from an instable conformation of the “Y”-structure mimicking a full-size antibody. We hypothesize that the oligomerization of Fc-fusion leads to physical instability, which can be overcome by disrupting dimerization. Therefore, we engineered the Fc by mutating critical residues located on the dimerization interface. This strategy allowed the expression of fully stable EPO monomeric Fc fusion proteins. Monomeric Fc-fusions can provide several advantages over traditional dimeric Fc-fusion technology. For example, many Fc monomer fusion proteins have enhanced biological activity in comparison with traditional dimeric Fc-fusions, presumably in part due to a reduction of interference between the effector proteins which can occur in dimeric Fc-fusions (Dumont et al., 2006). Importantly, small monomeric Fc can be exploited to expand the Fc-based therapeutic applications, due to its unique receptor binding pattern. Monomeric Fc (i) binds to FcRn and exhibits a similar *in vivo* half-life to wild-type dimeric Fc; (ii) lacks binding to FcγRIIIa, which results in the absence of Fc-effector functions; and (iii) possesses high-affinity binding to FcγRI that can be used for toxin targeting (Ying et al., 2012, 2014; Wang et al., 2017).

## Materials and Methods

### Cloning of EPO-Fusions

Erythropoietin-fusion sequences were amplified by PCR using as templates the *N. benthamiana* codon-optimized sequences of EPO (Jez et al., 2013) and rituximab IgG1 (HC, Acc. No. AX556949 and LC, Acc. No. AX556921). All primers used for cloning are listed in Supplementary Table 1.

#### Erythropoietin Fused to hIgG1-Fc

**FIGURE 1:**
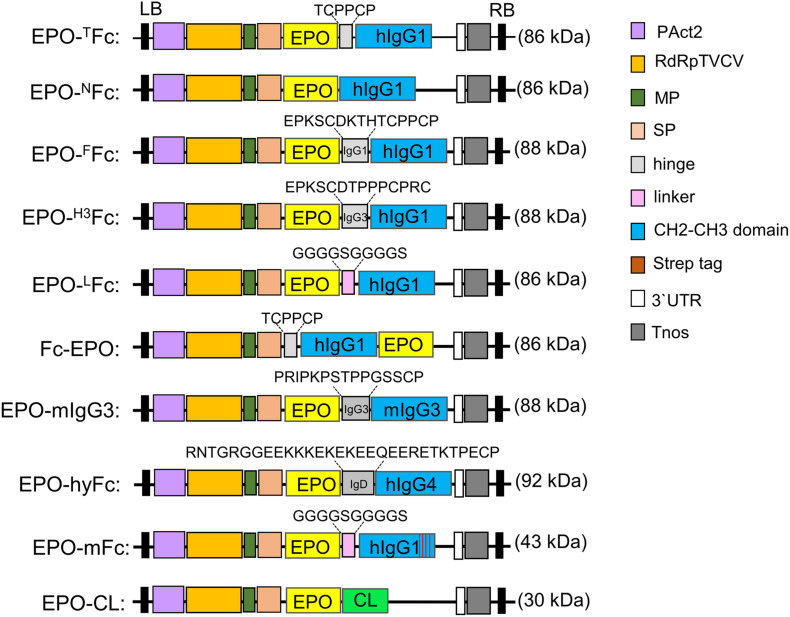
Schematic representation of the expression vectors used in this investigation. The modified TMV-based MagnICON vector (pICHα26211α) with a targeting sequence for the secretory pathway was used to clone the fusion proteins. Common features are: PAct2: Arabidopsis actin 2 promoter; RdRpTVCV, RNA-dependent RNA polymerase from Turnip vein clearing virus; MP, movement protein from the tobacco mosaic virus; SP, barley α-amylase signal peptide for apoplast targeting. Tnos, nopalin synthase gene terminator; 3′UTR, TVCV Pol. 3′-untranslated region; LB, left border; RB, right border; EPO, human erythropoietin; CL, constant domain of IgG1 light chain. The calculated protein molecular weight is shown for each native fusion (for full peptide sequence, see also Supplementary Figure 1).

Previously, we reported on the expression of EPO-Fc (EPO-^*T*^Fc) containing the EPO sequence lacking the signal peptide (amino acids 28–194) fused to a truncated hinge region and the CH2-CH3 domains of the human IgG1 (amino acids 248–470) (Castilho et al., 2011b). Here EPO-Fc variants at the hinge region were obtained by overlap extension polymerase chain reaction (OE-PCR). First, the EPO and Fc fragments (aa 254–470) were amplified using the primer pairs EPO F1/EPO R1 and Fc F1/Fc R1, respectively. The two fragments were used as templates for OE-PCR with primers EPO F1/Fc R1 and cloned into pICH26211α. This vector was used for the expression of EPO fused to a Fc fragment lacking a hinge region (EPO-^*N*^Fc, Figure 1 and Supplementary Figure 1). Similarly, the EPO and Fc fragments (aa 238–470) were amplified using primer pairs EPO F1/EPO R2 and H1 F1/Fc R1, respectively. After OE-PCR with primers EPOF1/Fc R1, the fragment was cloned into pICH26211α. This vector was used for the expression of EPO fused to a Fc fragment containing the full genetic hinge region (aa 216–230, EPKSCDKTHTCPPCP) of the human IgG1 (EPO-^*F*^Fc, Figure 1 and Supplementary Figure 1).

In another vector, we substituted the hinge of human IgG1 for a 15-residue segment of human IgG3 hinge region, rich in proline residues. The EPO fragment was amplified using primers EPOF1/EPO R3, and the Fc fragment was amplified with primers H3 F1/Fc R1 that modify the amino acids in the hinge region of EPO-^*F*^Fc. After OE-PCR with EPO-F1/Fc R1 primers, the EPO-Fc fragment was cloned into pICH26211α. This vector was used for the expression of EPO fused to a Fc fragment containing one repeat (EPKSCDTPPPCPRC) of the human IgG3 hinge region (EPO-^*H*3^Fc, Figure 1 and Supplementary Figure 1).

The EPO was also cloned with a Fc fusion at the N-terminus. cDNA encoding IgG1 Fc (aa 248–470) was amplified with primer pair Fc-EPO F1/Fc-EPO R1, while primers Fc-EPO F2/Fc-EPO R2 were used to amplify the EPO fragment (aa 28–194). OE-PCR was performed using Fc-EPO F1/Fc-EPO R2 primers, and the Fc-EPO fusion was cloned into pICH26211α vector. This vector was used for the expression of EPO with N-terminal Fc fragment containing the TCPPCP core motif of the hinge region and the CH2-CH3 domains of the human IgG1 (Fc-EPO, Figure 1 and Supplementary Figure 1).

Finally, we produced an EPO-Fc variant where the hinge region was substituted by a flexible polypeptide composed of glycine and serine residues. First, a CH2-CH3-Fc fragment (aa 258–470) with a “GS” linker at the N-terminus and with flanking *Eps*3I sites was amplified with primer pair LFc F1/Fc R2 introducing two internal *Bsa*I sites. This fragment was cloned into pICH26211α digested with *Bsa*I resulting in a cloning vector pICH26211α:^*L*^Fc (non-fused Fc). The EPO fragment, amplified with primer pair EPO F1/EPO R4 was cloned into *Bsa*I sites of pICH26211α:^*L*^Fc. This vector was used for the expression of the EPO and Fc domains separated by (Gly-Gly-Gly-Gly-Ser)_2_ flexible linker (EPO-^*L*^Fc, Figure 1 and Supplementary Figure 1).

#### Erythropoietin Fused to IgG3-Fc

The EPO fragment (aa 28–194) was also fused to IgG3 Fc from mouse IgG3 (aa 170–401, Acc. No. AHM27283). The Fc region was amplified with primers mIgG3 F1/mIgG3 R1, and after digestion with *Eps*3I, the DNA fragment was ligated to *Bsa*I-digested pICH26211α to produce the intermediate pICH26211α:mIgG3Fc. The two internal *Bsa*I sites introduced in this vector were used to clone in the EPO fragment amplified with primers EPO F1/EPO R5. This vector was used for the expression of EPO fused to the hinge-CH2-CH3 domains of the mouse IgG3 (EPO-mIgG3Fc, Figure 1 and Supplementary Figure 1).

#### Erythropoietin Fused to Hybrid Fc

A hybrid Fc fragment consisting of 30 aa (133–162) of the C-terminal IgD hinge region, 8 aa (SHTQPLGV; 163–170) of the N-terminal IgD CH2 domain, 100 aa (121–220) of the IgG4-CH2 domain, and 107 aa (221–327) of the IgG4-CH3 domain was synthesized and codon-optimized for *N. benthamiana*. The fragment carries two flanking *Eps*3I sites with two internal *Bsa*I sites. The *Eps*3I-digested fragment was cloned into pICH26211α digested with *Bsa*I, resulting in a cloning vector pICH26211α:hyFc. Next, the EPO fragment (aa 28–194) was amplified with primer pair EPO F1/EPO R6 and cloned into *Bsa*I sites of pICH26211α:hyFc. This vector was used for the expression of EPO fused to a hybrid IgD-IgG4 Fc (EPO-hyFc, Figure 1 and Supplementary Figure 1).

#### Erythropoietin Fused to Monomeric IgG1-Fc

A monomeric version of ^*L*^Fc (mFc) was generated by mutating critical residues on the IgG1-Fc dimerization interface located in CH3 domain (T366R, L368H, P395K, and F405R) (Ying et al., 2012; Wang et al., 2017).

First, a codon-optimized DNA fragment coding for an IgG1-CH3 domain (aa 364–470) carrying the four mutations was synthesized. A CH2-CH3-Fc fragment (aa 258–470) with a “GS” linker at the N-terminus flanked by *Eps*3I sites was generated by OE-PCR. The CH2 domain was amplified with primer pair LFc F1/mFc R1, and primers mF1 F1/CH3 R1 were used to amplify the mutated CH3 domain. The mFc was assembled by OE-PCR with primers LFc F1/CH3 R1. The *Eps*3I-digested fragment was cloned into pICH26211α digested with *Bsa*I, resulting in a cloning vector pICH26211α:mFc (non-fused monomeric Fc). Finally, the EPO fragment, amplified with primer pair EPO F1/EPO R4, was cloned into the *Bsa*I sites of pICH26211α:mFc. This vector was used for the expression of the EPO fused to a monomeric Fc with a (G4S)_2_ flexible linker (EPO-mFc, Figure 1 and Supplementary Figure 1).

#### Erythropoietin Fused to IgG-Lc

First, the constant region of the IgG1 light chain (CL) (aa 129–235) was amplified with primers CL F1/CL R1 with two *Bsa*I cloning sites on N-terminus, as described above. After digestion with *Eps*3I, the fragment was cloned into *Bsa*I sites on pICH26211α to generate the cloning vector pICH26211α:CL. EPO was amplified with EPO F1/EPO R5 primers and cloned into pICHα26211α:CL vector. This vector was used for the expression of EPO fused to the constant region of the human IgG1 light chain (EPO-CL, Figure 1 and Supplementary Figure 1).

### Plant Material and Analysis of Protein Expression

*Nicotiana benthamiana* ΔXTFT plants were grown in a growth chamber at 22°C with a 16-h light/8-h dark photoperiod. All fusion proteins and antibodies or antibody fragments were transiently expressed *via* agro-infiltration. Total soluble proteins (TSP) and proteins secreted to the apoplastic fluid (AF) were collected 4–5 days post-infiltration (Kriechbaum et al., 2020). Expression of EPO-fusions was analyzed by immunoblotting of SDS–PAGE or native PAGE using mouse anti-EPO (1:5000, MAB2871, R&D Systems, Minneapolis, MN, United States), anti-human IgG-HRP (HC and LC, 1:10,000, W4031, Promega, Mannheim, Germany), anti-mouse IgG-HRP (1:10,000, Sigma-Aldrich, A0545), and anti-human kappa-chain-HRP (LC, 1:1000, Sigma-Aldrich, A7164) antibodies. EPO-Fc variants were purified from agroinfiltrated leaves (800 mg) with rProtein A or Protein G Sepharose^TM^ Fast Flow (GE Healthcare) and with KappaSelect^TM^ (GE Healthcare) for EPO-CL, according to the manufacturer’s instructions. Heavy chains of cetuximab (Castilho et al., 2015), anti-ebola 13F6 (Castilho et al., 2011a) antibodies, and 2G12-ScFvFc (Loos et al., 2011) were cloned previously, and their expression was analyzed with anti-human gamma-chain-HRP (HC, 1:1000, Sigma-Aldrich, A7164). All purified proteins were stained with Coomassie Brilliant Blue.

### Glycan Analysis and Peptide Mapping

Site-specific *N-*glycosylation profiling of recombinant proteins was determined using the reverse-phase liquid chromatography–electrospray ionization mass spectrometry (LC-ESI-MS) of tryptic glycopeptides as described previously (Grunwald-Gruber et al., 2017). Identification of *N*- and *C-*termini was made by tryptic peptide mapping using LC-ESI-MS/MS (Pabst et al., 2012).

## Results

### Erythropoietin Fusions to Human IgG1-Fc Are Instable When Expressed in Plants

**FIGURE 2:**
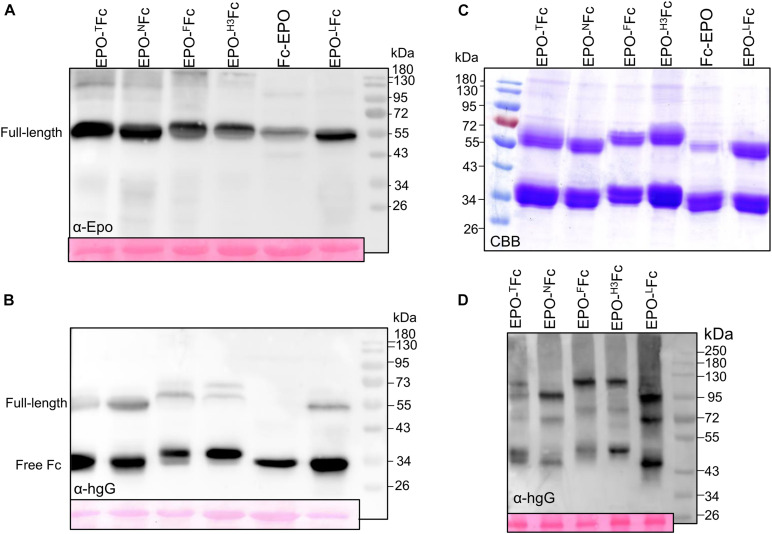
Analysis of the expression of EPO-IgG1 variants. Total soluble protein (TSP) extracts from *N. benthamiana* ΔXTFT leaves expressing different EPO-Fc variants were analyzed by immunoblotting at 5 days post-infiltration (dpi) using **(A)** anti-EPO and **(B)** anti-hIgG antibodies. **(C)** EPO-Fc variants purified out of TSP with protein A were stained with Coomassie Brilliant Blue under reducing conditions (CBB). **(D)** Oligomerization of EPO-Fc variants was evaluated in 8% native PAGE of TSP non-reducing samples. Ponceau staining shows similar amounts of protein loaded. Protein size markers are shown in kilo Dalton (kDa).

Previously, we reported on the expression of EPO-Fc containing the EPO sequence fused to the central polyproline core of the hinge region of a human IgG1 (Castilho et al., 2011b). We showed that the fusion protein (EPO-^*T*^Fc, Figure 1 and Supplementary Figure 1) is not stable when expressed in *N. benthamiana* and high levels of free Fc domain are detected by Western blots using anti-IgG antibodies (Figures 2A,B). After Protein A purification, the full-size fusion protein (∼55 kDa) and the free Fc (∼34-kDa) are stained in Coomassie gels under reducing conditions (Figure 2C). It is well known that the hinge region of monoclonal antibodies is prone to proteolytic degradation (Niemer et al., 2014). In this investigation, we have altered the hinge region to produce EPO-Fc fusions (i) without a hinge region (EPO-^*N*^Fc, Figure 1 and Supplementary Figure 1), (ii) with the full hinge region of the human IgG1 (EPO-^*F*^Fc, Figure 1 and Supplementary Figure 1), and (iii) with a segment of the hinge region of human IgG3 rich in proline residues (EPO-^*H*3^Fc, Figure 1 and Supplementary Figure 1). In addition, we evaluated the expression of EPO fused to an IgG1-Fc at the N-terminus (Fc-EPO, Figure 1 and Supplementary Figure 1). We have also replaced the processing-prone part of the hinge region with a protease-resistant linker known to improve the protein folding and stability of fusion proteins (Chen et al., 2013). We designed a construct to express EPO and Fc domains separated by a flexible linker sequence consisting of stretches of glycine (Gly) and serine (Ser) residues (“GS” linker). The (Gly-Gly-Gly-Gly-Ser)_2_ sequence was inserted between the EPO and CH2-CH3 domains to provide flexibility, and allow for mobility of the connecting domains (EPO-^*L*^Fc, Figure 1 and Supplementary Figure 1). Western blot analysis of TSP and subsequent protein A purifications show that all EPO-Fc variants are not stably expressed in *N. benthamiana* and large amounts of free Fc are detected (Figures 2A–C). In detail, an analysis of TSP with anti-EPO antibodies reveals the expression of the full-length fusion (∼55 kDa), and faint bands at ∼35 kDa correspond to EPO fragment (Figure 2A). Anti-IgG antibodies also detect the full-length protein (∼55 kDa) and a strong band at ∼34 KDa corresponding to the free Fc (Figure 2B). For Fc-EPO variant, anti-EPO antibodies are able to detect the presence of full-length fusion, which is not detected with anti-IgG antibodies when the same amount of TSP from all fusions is loaded on the gel (Figure 2B). This result shows that compared with other fusions, the Fc-EPO variant is the most unstable. All EPO fusions purified with protein A are stained as a 55-kDa band along with the free Fc under reducing conditions (Figure 2C). The fact that both the full-length fusions and the free Fc have a higher molecular weight than expected (∼44 KDa and ∼26 KDa, respectively) is due to glycosylation (Castilho et al., 2011b, 2012, 2013). All EPO-fusions are secreted to the apoplast as the analysis of the AF with anti-EPO antibodies detects the full-size proteins (Supplementary Figure 2).

Analysis of EPO-Fc fusions in native PAGE revealed the presence of three bands. In EPO-^*N*^Fc and EPO-^*L*^Fc, lacking the hinge region, homodimers of EPO-Fc and of free Fc are detected at ∼100 and ∼43 kDa, respectively, while the band at 72 kDa should reflect the presence of heterodimers (EPO-Fc/Fc, Figure 2D). For EPO-^*F*^Fc and EPO-^*H*3^Fc, these three bands are slightly heavier (∼120, 75, and 45 kDa, Figure 2D). The discrepancy of the molecular weights compared to the calculated mass for dimers is probably due to protein folding. Indeed, analysis of the expression of free Fc (non-fused protein, ^*L*^Fc) shows the presence of 34 KDa in reducing samples and of 43 kDa in native conditions (Supplementary Figure 3).

### Alternative Fc Domains Do Not Increase the Stability of EPO-Fusions

Previous studies have shown that Fc chains from a different origin other than humans lead to an increase in the accumulation levels of nanobodies (VHH)-Fc fusions targeted to the ER of *Arabidopsis thaliana* and that the Fc chain determines the amount of cleavage in the C-terminal part of the nanobody (De Buck et al., 2013). To investigate whether the origin of the Fc domain has an influence on the accumulation level and the stability of EPO-Fc fusions, we designed EPO-fusions using the hinge-CH2-CH3 domains of mouse origin (EPO-mIgG3, Figure 1 and Supplementary Figure 1).

In addition, other studies have evaluated the performance of EPO fused to a natural form of highly flexible hybrid Fc (hyFc). hyFc was engineered by replacing the hinge and the upper N-terminal of CH2 domains of human IgG4 Fc (essential for binding to human FcγRs) with the corresponding regions of IgD, which shows the highest hinge-fold flexibility (Roux et al., 1998). EPO fused with hyFc retained “Y-shaped” structure and achieved better *in vitro* and *in vivo* bioactivity than EPO-IgG1 Fc, possibly owing to the flexible hinge region (Im et al., 2011). To investigate if the highly flexible hinge region of IgD could increase the accumulation of full-length EPO-Fc fusions, we designed a vector to express EPO-hyFc in *N. benthamiana* (Figure 1 and Supplementary Figure 1).

**FIGURE 3:**
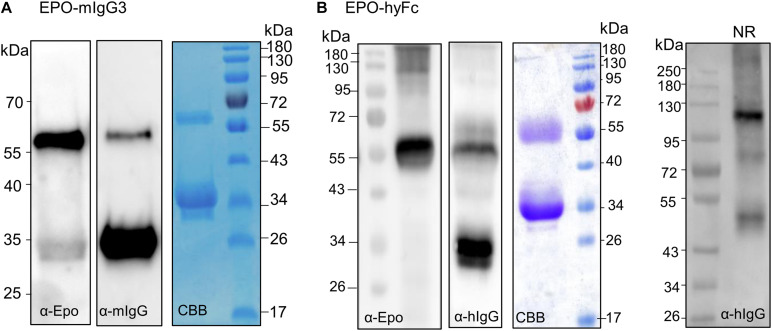
Analysis of the expression of EPO-IgG3 and EPO-HyFc. **(A)** Total soluble protein (TSP) extracts from *N. benthamiana*ΔXTFT leaves expressing EPO-mIgG3 were analyzed by immunoblotting with anti-EPO and anti-mouse IgG antibodies. EPO-mIgG3 was purified out of TSP with protein G and stained with Coomassie Brilliant Blue under reducing conditions (CBB). **(B)** TSP from *N. benthamiana*ΔXTFT leaves expressing EPO-hyFc were analyzed by immunoblotting with anti-EPO and anti-IgG. Protein A purified proteins were stained with Coomassie Brilliant Blue under reducing conditions (CBB). Oligomerization of EPO-hyFc was evaluated in 8% native PAGE of TSP non-reducing samples. Protein size markers are shown in kilo Dalton (kDa).

Erythropoietin-mIgG3 and EPO-hyFc were transiently expressed in *N. benthamiana*, and expression was determined by Western blotting using anti-EPO and anti-IgG antibodies. Also, fusion proteins were purified with protein G (EPO-mIgG3) or protein A (EPO-hyFc). The results showed once again that full-length fusion proteins are not stable and result in large amounts of free Fc (Figure 3). Anti-EPO antibodies detect the presence of full-length fusions, while anti-IgG antibodies detect both full-length (∼55-kDa) and free Fc (∼34-kDa) (Figures 3A,B). Comparing other EPO-human IgG1 fusions, an analysis of the same amount of TSP shows that EPO-mIgG3 is expressed at a much lower level (Figure 3A), while EPO-hyFc is expressed at a similar or slightly increased level (Figure 3B). In addition, native PAGE of TSP shows that EPO-hyFc dimerization is similar to that of EPO-^*F*^Fc and EPO-^*H*3^Fc (Figure 3B).

### Monoclonal IgG Fragments Are Instable When Expressed in *N. benthamiana*

**FIGURE 4:**
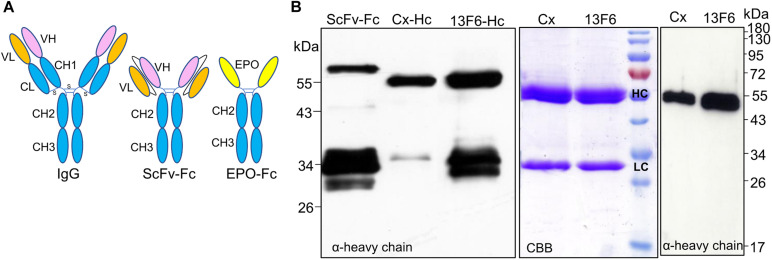
Analysis of the expression of IgG1 fragments. **(A)** Schematic structures of a monoclonal antibody, a ScFv-Fc and of EPO-Fc. **(B)** Total soluble protein (TSP) extracts from *N. benthamiana*ΔXTFT leaves expressing ScFv-Fc and the heavy chain (HC) of IgG1 monoclonal antibodies cetuximab (Cx) and 13F6 were analyzed by immunoblotting with anti-γ chain antibodies (HC of IgG1). Full cetuximab and 13F6 antibodies (HC and LC) were purified out of TSP with protein A and stained with Coomassie Brilliant Blue under reducing conditions (CBB). Western blotting with anti-γ-chain antibodies detects only the HC of the Cx and 13F6 antibodies. Protein size markers are shown in kilo Dalton (kDa).

Production of Fc fusions or full-length antibodies in plants often results in a significant amount of degradation products (De Muynck et al., 2010; Niemer et al., 2014). Also, antibodies with an engineered single-chain variable fragment (ScFv) are prone to degradation when expressed in tobacco-related species (Loos et al., 2011; Catellani et al., 2020). These molecules consist of a ScFv fused to the Fc of an antibody (hinge-CH2-CH3) and spontaneously folded and dimerized to give a “Y” structure similar to a full-length IgG (Zhang et al., 2007). Although EPO-Fc fusions also mimic a “Y”-shape (Im et al., 2011) and are able to dimerize, the lack of the CH1 domain and the light chain interaction seemingly foster the breakage of full-length fusion proteins (Figure 4A). To further investigate the possible involvement of (the lack of) light chain or CH1 region in the protein instability, we expressed the heavy chain of two monoclonal antibodies cetuximab (Castilho et al., 2015) and 13F6 (Castilho et al., 2011a) and an antibody fragment, ScFv-Fc (Loos et al., 2011), in *N. benthamiana* leaves (Supplementary Figure 1). After Protein A purification, samples were analyzed by Western blotting using anti-heavy chain antibodies. The full-length ScFv-Fc protein was detected at 60 kDa but also a strong signal is visible at ∼34 kDa (Figure 4B). Similar results were obtained when the 13F6-Hc was expressed without its light chain. Full-length heavy chain was detected at 55 kDa but ∼34-kDa fragments reacting with anti-heavy chain antibodies were also detected. The heavy chain of cetuximab seems to be more resistant to degradation although the 34-kDa fragment is present (Figure 4B). Degradation is not detected when the heavy chain of both cetuximab and 13F6 is co-expressed with the corresponding light chain (Figure 4B). Coomassie Brilliant Blue staining under reducing conditions shows both heavy (HC) and light chains (LC) but only HC react to anti-heavy chain antibodies (Figure 4B). These results seem to point out the importance of a balanced expression of HC and LC and of the disulfide bonds in the CH1-CL interface for the stability of IgGs.

### N-Terminus Identification of the Fc Degradation Product Reveals No Consensus Sequence

Mass spectrometry (MS) analysis of the 34-kDa protein (free Fc) was used to identify its N-terminus. Potential cleavage sites identified for some constructs showed that they are not sequence-specific and occurred within the C-terminal end of the EPO sequence and within the hinge region (Table 1). Identification of the N-terminus of selected fusion proteins cannot pinpoint a particular cleavage site, which indicates that the degradation of plant-derived Fc fusions is not solely due to specific enzymatic activity.

**TABLE 1:**
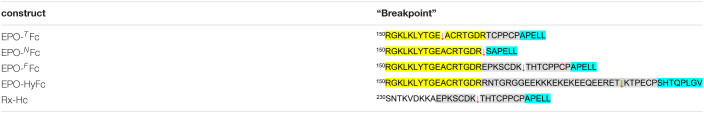
Fusion “breaking points.”

*Hinge sequence is in gray, EPO sequence in yellow, and Fc in blue. Peptides upstream the (↓) were not found (see also Supplementary Figure 1 for full peptide sequences).*

### Monomeric EPO-Fc Fusion Proteins Are Stable in Plants

Dimerization of Fc occurs not only through disulfide bridges in the hinge but also by strong non-covalent interactions between the two CH3 domains. Therefore, all EPO-Fc fusions (IgG1, IgG3, and IgG4) described above should mimic the antibody “Y”-shape of a full antibody but without interactions with light chains.

We hypothesize that monomeric Fc fusions might reduce the interference between the effector proteins, which can occur in dimeric Fc-fusion proteins.

Monomeric Fc fusions consist of only one effector molecule and one Fc monomer, and thus, the size is largely reduced compared to previous monomeric Fc fusions where a single effector function molecule was fused to a dimeric Fc. These novel mFc are relatively stable and also represent the smallest fragments (∼27-kDa) of IgG that retain binding to FcRn comparable with that of wild-type Fc (Ying et al., 2012).

Previous studies have generated a large phage library of IgG1 Fc molecules with extensive mutations in the dimerization interface and identified specific mutations (S351L, T366R, L368H, and P395K) essential to produce soluble monomeric Fc (mFc) (Ying et al., 2012). Also, a F405R mutation seems to favor Fc monomer formation (Shan et al., 2016). Later, it was shown that the S351L mutation might contribute to the non-specific binding of mFc to unrelated targets (Ying et al., 2012; Wang et al., 2017).

Here, we set up to express EPO fusion to a mFc (EPO-mFc) by introducing four mutations (T366R, L368H, P395K, and F405R) in the CH3 domain of EPO-^*L*^Fc.

**FIGURE 5:**
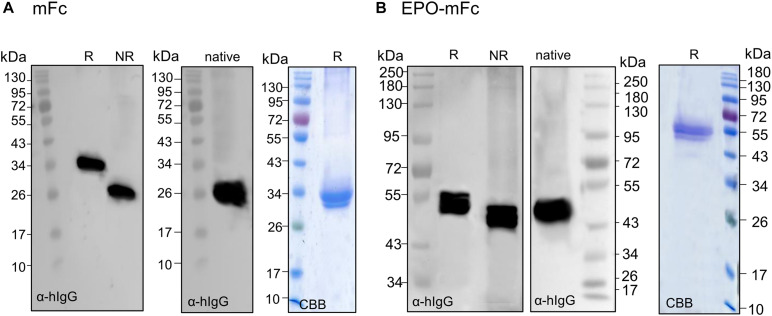
Analysis of the expression of monomeric EPO-mFc fusions. Total soluble proteins (TSP) extracts from *N. benthamiana*ΔXTFT leaves expressing mFc (non-fused) were analyzed in 12% SDS–PAGE under reducing (R) and non-reducing (NR) conditions and in native PAGE with anti-hIgG antibodies. **(A)** mFc purified out of TSP with protein G was stained with Brilliant Blue under reducing conditions (CBB). **(B)** EPO-mFc expression was analyzed in reduced (R) and non-reduced (NR) TSP extracts in 8% SDS–PAGE and in native 12% PAGE. EPO-mFc was purified out of TSP with protein G and stained with Coomassie Brilliant Blue under reducing conditions (CBB). Protein size markers are shown in kilo Dalton (kDa).

To ensure that the mutations at CH3 domain disturb Fc dimerization, we first expressed mFc (non-fused) and analyzed the expression under reducing and non-reducing conditions. Contrasting with dimeric ^*L*^Fc (Supplementary Figure 3), immunoblotting with anti-IgG antibodies reveals the presence of a single band at ∼34 kDa in reducing PAGE and at ∼26 kDa in native PAGE (Figure 5A). Next, we analyzed TSP expressing EPO-mFc with anti-IgG antibodies. The results show that the protein is detected as a single band (∼55 kDa) under reducing conditions, and no significant degradation products were detected. As for mFc, in non-reducing native conditions, EPO-mFc is detected at a slightly lower molecular size most probably due to protein folding (Figure 5B). EPO-mFc was purified out of TSP with protein G since it was shown that mFc has an increased binding affinity to protein G compared to protein A (Ying et al., 2013). In contrast to dimeric EPO-^*L*^Fc, EPO-mFc shows a single protein band at the expected size for the full-length protein (∼55-kDa) (Figure 5B).

### Fusions to Lc Domains Are Stable and Provide a New Protein Purification Tag

**FIGURE 6:**
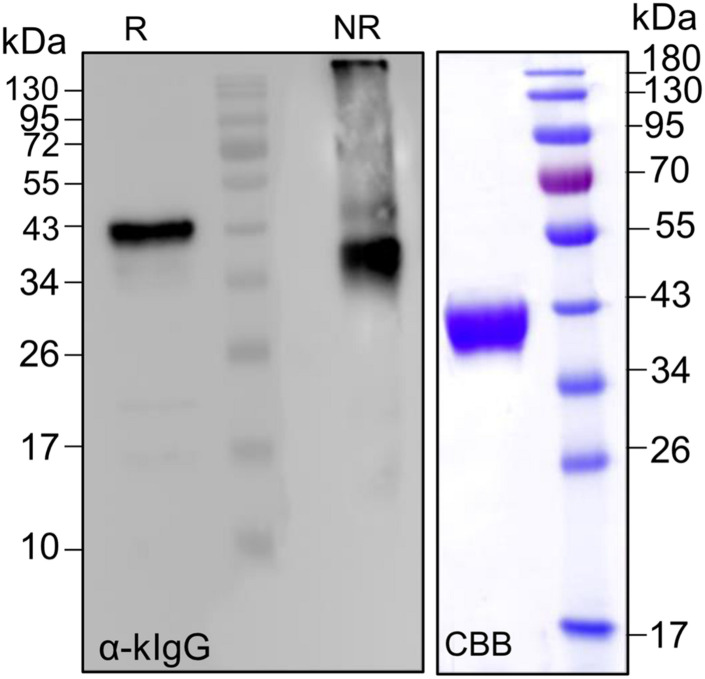
Analysis of the expression of EPO-CL fusions. Total soluble proteins extracted from *N. benthamiana*ΔXTFT leaves expressing EPO-CL were analyzed with anti-kappa IgG antibodies (light chain) under reducing and non-reducing conditions. EPO-CL purified out of TSP with kappa-select resin was stained with Coomassie Brilliant Blue under reducing conditions (CBB). Protein size markers are shown in kilo Dalton (kDa).

Our previous attempts to purify EPO out of TSP showed that His and strep II tags are either cleaved off or not accessible. In addition, a newly established peptide tag (ELDKWA) was well suited for EPO purification but the recombinant protein was retained in the ER and decorated mainly with high mannosidic glycans (Jez et al., 2013). Here, we designed a construct for the expression of EPO fused to the constant domain of the IgG-light chain (CL). Analysis of TSP extracts with anti-EPO and anti-kappa chain antibodies shows that EPO-CL is expressed as a single protein corresponding to the full-length fusion (∼40-kDa) and no degradation is observed (Figure 6). Although free full-size light chains from immunoglobulins are known to form dimers, non-reducing samples of EPO fused to their constant domain do not show a size shift that could correspond to the formation of dimers (Figure 6). Importantly, kappa-select allows efficient purification of EPO-CL fusion (Figure 6).

### Monomeric EPO-Fusions Are Decorated With Complex N-Glycans

Erythropoietin is a ∼30-kDa glycoprotein with three N-linked glycosylation sites (Asn^24^, Asn^38^, and Asn^83^), and glycosylation accounts for about 40% of the total molecular weight. The Fc domain of IgGs contains a single conserved glycosylation site at the CH2 domain. We have previously reported the glycosylation profile of EPO-^*T*^Fc expressed in *N. benthamiana* glycosylation mutants ΔXTFT (Strasser et al., 2008). Additionally, we showed that we can modulate EPO glycosylation toward human-like complex N-glycosylation (Castilho et al., 2011b, 2013) and O-glycosylation (Castilho et al., 2012). All EPO-Fc fusion proteins produced in this investigation showed a similar glycosylation profile with mainly complex glycans (GnGn) devoid of core xylose and fucose and with additional high mannose (Man8 and Man9) and Lewis-A [Gn(FA)] carrying glycans (Castilho et al., 2011b; Jez et al., 2013; Supplementary Figure 4).

In addition, we analyzed the glycosylation profile of EPO-mFc and EPO-CL expressed in ΔXTFT. Glycan profiling shows that all sites on the EPO domain are decorated with complex human-like glycans (GnGn) devoid of plant-specific glyco-epitopes as described before (Supplementary Figure 4). This indicates that the stabilized fusion proteins are processed through the Golgi apparatus as for the dimeric EPO-Fc variants.

## Discussion

Plants have proven to be versatile production systems for the expression and assembly of heavy and light chains of functional antibodies. These include full-sized and chimeric IgG, IgA, IgE, Ig fragments (ScFv and Fab), and even complex molecules such as pentameric and hexameric IgMs (Loos and Steinkellner, 2018; Komarova et al., 2019). However, some reports on the expression of antibodies and antibody fragments in plants indicate that there are challenges regarding expression, subcellular localization, and proteolytic degradation. Although some antibodies undergo proteolytic degradation (De Muynck et al., 2010), it is difficult to draw a general rule for the degradation pattern because of the diversity of the host species, expression system, and antibodies tested. Most studies on the degradation of antibodies and antibody fragments pinpoint the antibody hinge and closely related regions as the most sensitive to proteolysis and proteolytic enzymes, owing to its non-compact, extended conformation (De Muynck et al., 2010; Niemer et al., 2014). Despite these reports, the degradation of plant-derived full antibodies is still an exception and not the rule. The same is not true for the expression of Fc-fusions where proteolysis is often observed in plants. Fc-fusions (also known as Fc chimeric fusion proteins) are composed of the Fc domain of an immunoglobulin genetically linked to a therapeutic protein of interest. The Fc domain is used as a supporting module to which proteins can be attached while retaining their native biological activity. The main reason for fusing proteins to the Fc domain is to enhance solubility and half-life of a therapeutic protein (Levin et al., 2015) and in addition, facilitating protein purification of recombinant protein by protein A chromatography (Carter, 2011). Successful therapeutic proteins that are recombinant Fc-fusions have become an important class of molecules in biotechnology, and many therapeutic Fc-fusions have been FDA-approved. We have previously expressed human EPO in *N. benthamiana* without (Jez et al., 2013) and with Fc-fusion (Castilho et al., 2011b, 2012, 2013; Nagels et al., 2012). EPO-Fc fusions are used for the pulmonary delivery of recombinant EPO to patients. The interaction of the Fc domain with Fc receptors on the epithelial cells of the lungs is exploited to transfer the EPO-Fc across the epithelium barrier to the blood stream (Bitonti et al., 2004). Plant-derived EPO-Fc was shown to be active *in vitro* (Nagels et al., 2012; Castilho et al., 2013) but compared to monoclonal antibodies, the achieved expression yields are extremely low. This low expression results from fusion instability since a large portion of the recombinant proteins (about 30–50 times) consist of Fc lacking the EPO fragment (free Fc). The presence of free Fc has already been reported for the expression of EPO-Fc in chicken (Penno et al., 2010) but not in mammalian cells (Salgado et al., 2015).

Here, we evaluated the degradation/instability of different EPO-Fc chimeras. We have manipulated the hinge region on the Fc domain (EPO-^*N*^Fc, EPO-^*F*^Fc, EPO-^*H*3^Fc, EPO-hyFc) or replaced it with peptide sequences more resistant to proteolysis (EPO-^*L*^Fc). We have also replaced the Fc domain from human IgG1 with mouse IgG3 (EPO-mIgG3), a strategy used to increase the accumulation of VHH in arabidopsis seeds (De Buck et al., 2013). The results showed no significant impact on stabilizing the expression of the full-length fusions. Although the degradation of Fc-fusion proteins by plant proteases is a plausible explanation, our results indicate that structural/conformation issues are also involved. Analysis of EPO-fusions under native conditions indicates that the recombinant proteins are multimeric (homo- and heterodimers). Indeed, the “Y-shape” conformation of Fc-fusions might lead to (i) steric hindrance between two functional domains, (ii) altered biodistribution and metabolism of the protein moieties due to interference with each other, or (iii) incorrect folding of the fusion protein (Yao et al., 2004).

The instabilities observed for Fc fusions are not restricted or due to the EPO-fusion partner. Many therapeutic proteins have been expressed in plants fused to the Fc domain, and to our knowledge, the majority of these studies have reported on the presence of degradation products derived from the Fc partner (Lu et al., 2012; De Buck et al., 2013; Kim et al., 2017, 2018; Rattanapisit et al., 2019; Xiong et al., 2019; Diamos et al., 2020; Castilho et al., 2021). The expression of the tumor-associated antigen (GA733) fused to the hinge-CH2-CH3 domain of human IgG1 (GA733-Fc) with and without a KDEL signal showed that ER retention increased the accumulation of the recombinant protein but large amounts of free Fc are detected (Lu et al., 2012). Similar results were observed for the expression of nanobodies (VHH) fused to human IgG1 expressed in arabidopsis seeds and retained in the ER (VHH-Fc). In this study, the protease sensitivity of VHH-Fc is influenced by the type of Fc chain, as less degradation occurred with the Fc chain of mouse IgG3 compared to human IgG1 (De Buck et al., 2013). However, different VHHs fused to mouse IgG3-Fc and expressed in arabidopsis seeds; *N. benthamiana* and *Pichia pastoris* showed some degree of degradation (De Meyer et al., 2015). Also, from several human IgG1 fusions to the Zika virus envelope domain III (ZE3), the ZE3-Fc fusion had particularly high levels of degradation (Diamos et al., 2020). Another study report on dengue Fc-fusion proteins where the consensus EDIII domain of dengue envelope protein was linked to CH2-CH3 domains of both mouse (IgG2a) and human (IgG1) by a short peptide derived partly from CH1 domain and partly from the hinge region (cEDIII-Fc). Expression of cEDIII-Fc variants in *N. benthamiana* plants reveals a mixture of dimers and polymers as well as free Fc described as truncated versions (Kim et al., 2017).

Flexible linkers are usually applied when the joined domains require a certain degree of movement or interaction. In the fusion of osteopontin (OPN) to the human IgG1 Fc domain (OPN-Fc), the two domains were separated by a flexible (Gly4Ser)_3_ linker to ensure the correct protein folding and function. Analysis of OPN-Fc expression with anti-OPN antibodies detects significant amounts of free OPN. Unfortunately, SDS–PAGE analysis with anti-IgG antibodies excluded the expected size for free Fc (Rattanapisit et al., 2019). A similar linker was used to separate the angiotensin-converting enzyme 2 (ACE2) and Fc-IgG1 domains (ACE2-Fc) as described here for EPO-^*L*^Fc. Expression of ACE2-Fc in *N. benthamiana* also shows the accumulation of free Fc (Castilho et al., 2021).

Instability is also observed for fusions to other immunoglobulins. Expression of VHH-IgA-based monomeric (bivalent), dimeric (tetravalent), and secretory IgA (tetravalent) fusions in Arabidopsis shows a high degree of protein instability (Virdi et al., 2013).

It is clear from the results presented in this investigation and from many reports in the literature that there are limitations in the production of Fc fusion proteins in plants. Even in mammalians, therapeutic fusion proteins may experience poor expression as the fusion partners interfere with each other for optimal translation (Leader et al., 2008; Robert et al., 2009; Chakrabarti et al., 2016).

What determines the degree of degradation is still the subject of research but most probably it is a combination of several factors. Although the hinge-Fc region influences the 3D structure and surface exposure of protease-sensitive cleavage sites (De Muynck et al., 2010), it also seems that non-covalent interactions and the interchain disulfide bond with the light chain stabilize the “Y” structure of monoclonal antibodies. We and others have shown that ScFv-Fc expressed in plants is equally prone to degradation (Loos et al., 2011). This recombinant protein mimics the confirmation of full-size antibodies lacking the light chain. Expression of heavy chains without the corresponding light chain also leads to breakage within the hinge region. Cetuximab and 13F6 antibodies are fully assembled and stably expressed in *N. benthamiana* but the expression of individual heavy chains resembles the expression of Fc-fusion proteins and ScFv-Fcs. It is possible that the degradation observed for some antibodies expressed in plants (De Muynck et al., 2010) is due to differences in the ratio of expression between heavy and light chains favoring the heavy chain and thus resulting in incorrect HC-LC pairing. This highlights the importance of disulfide bonds in the CH1-CL interface for the stability of IgG and IgG-derived fusion proteins.

It seems that in plants, the applicability of the traditional Fc fusion technology is largely hampered by its dimeric nature and, in this configuration, steric hindrance between therapeutic protein domains might lead to physical instability. We hypothesized that the disruption of Fc dimerization can be one strategy to produce stable fusion proteins.

Dimerization of hinge less Fcs is mediated by a large hydrophobic interface in its CH3 domain, involving at least 16 residues in each polypeptide chain that make intermolecular interactions (Ying et al., 2014; Wang et al., 2017).

“Monomeric” technology is an upgraded version of the traditional dimeric Fc fusions. Monomeric Fcs (mFc) are highly soluble and retained binding to human FcRn showing that dimerization is not required for effective receptor binding (Wang et al., 2017). mFc provides several advantageous features compared to dimeric Fc such as (i) the smaller size that can enable better tissue penetration particularly essential for use in cancer therapy, (ii) no effector functions since for most therapeutic proteins direct cytotoxicity is not involved in their mode of action and in many cases should be avoided (Dimitrov, 2012), and (iii) the unique FcγR-binding profile leading to improved therapeutic efficacy in some clinical applications (Ying et al., 2014). Also, the loss of multivalence in mFc fusions is not always a disadvantage. For example, particular signaling pathways, such as receptor tyrosine kinases, require monovalent targeting to avoid receptor agonism caused by receptor dimerization from bivalent antibodies or Fc fusions (Merchant et al., 2013).

Previous studies have engineered monomeric Fcs by mutating four to seven critical residues located on the IgG1-Fc dimerization interface. One of the identified Fc monomers has only four mutations to the wild-type IgG1 Fc and is half the size (S351L, T366R, L368H, and P395K) (Ying et al., 2012, 2014).

Here, we generated a mFc by introducing four mutations in the CH3 domain (T366R, L368H, P395K, and F405R). Mutation of the serine residue (S351L) was excluded since in a follow-up study, this mutation was found to be involved in the binding of mFc to unrelated proteins (Wang et al., 2017). A mutation at F405 was also introduced based on previous work showing Fc had favored monomer formation when F405 was mutated to R, Q, or E (Shan et al., 2016). Transient expression of the mFc in *N. benthamiana* showed that the protein is no longer able to dimerize. Importantly, when EPO is fused to the mFc, the monomeric fusion protein is stable and no degradation products are detected.

Altogether, it seems that the transient expression of EPO-mFc *N. benthamiana* is promising and warrants the further development of monomeric Fc-based fusion proteins in plants.

Finally, in the course of this investigation, we identified a suitable tag for recombinant protein expression and purification. Fusions to the constant domain of the IgG light chain (CL) are stable and can be used as alternatives to His, StrepII, or FLAG tags to assess recombinant protein expression (with anti-light chain antibodies) and purification (with kappa select resins).

## Data Availability Statement

The original contributions presented in the study are included in the article/Supplementary Material, further inquiries can be directed to the corresponding author.

## Author Contributions

AC designed the experiments and wrote the manuscript. PG, SI, SK, and AC conducted the experiments. CG-G performed glycan analysis experiments. AC, SI, CG-G, and PG analyzed the results. All authors have made a substantial and intellectual contribution to the work and approved it for publication.

## Conflict of Interest

The authors declare that the research was conducted in the absence of any commercial or financial relationships that could be construed as a potential conflict of interest.

## Supplementary Material

Supplementary Figure 1 Peptide sequence of the proteins analyzed in this investigation.

Supplementary Figure 2 Analysis of the expression EPO-IgG1 variants in the apoplast.

Supplementary Figure 3 Analysis of the expression ^*L*^Fc (non-fused).

Supplementary Figure 4 Site-specific *N*-glycosylation profiles.

Supplementary Table 1 Primers used in PCR for gene cloning and construction of EPO-fusion variants.
