# Supplementary material for: The Instability of Dimeric Fc-Fusions Expressed in Plants Can Be Solved by Monomeric Fc Technology
Source: Front Plant Sci. 2021 Jul 9;12:671728. doi: 10.3389/fpls.2021.671728 (PMC8299721; doi:10.3389/fpls.2021.671728)

Table S1: Primers used in PCR for gene cloning and construction of EPO-IgG variants. *Bsa*I (GGTCTC) and *Eps*3I (CGTCTC) restriction sites are in bold.

Construct	Primers
EPO-^{T}Fc	EPO F1: GCA**GGTCTC**AAGGTGCTCCACCACGTCTTATC
	Fc R1: GCA**GGTCTC**AAAGCTCACTTTCCAGGAGAAAGAG
EPO-^{N}Fc	EPO F1: GCA**GGTCTC**AAGGTGCTCCACCACGTCTTATC
	EPO R1: CGTACTGGCGATCGTACATGCCCACCGTG
	Fc F1: CACGGTGGGCATGTACGATCGCCAGTACG
	Fc R1: GCA**GGTCTC**AAAGCTCACTTTCCAGGAGAAAGAG
EPO-^{F}Fc	EPO F1: GCA**GGTCTC**AAGGTGCTCCACCACGTCTTATC
	EPO R2: CAAGATTTGGGCTCACGATCGCCAGTACG
	H1 F1: CGTACTGGCGATCGTGAGCCCAAATCTTG
	Fc R1: GCA**GGTCTC**AAAGCTCACTTTCCAGGAGAAAGAG
EPO-^{H3}Fc	EPO F1: GCA**GGTCTC**AAGGTGCTCCACCACGTCTTATC
	EPO R3: GGTGCGCACCGTGGGCATGGGGGAGGTGTGTCACAAGATTTG
	H3 F1: CAAATCTTGTGACACACCTCCCCCATGCCCACGGTGCGCACC
	Fc R1: GCA**GGTCTC**AAAGCTCACTTTCCAGGAGAAAGAG
EPO-^{L}Fc	LFc F1: GAG**CGTCTC**TAGGTA**GAGACC**GGGATCC**GGTCTC**TGGAGGTGGTGGTTCTGGTGGTGGTGGTTCACTTGGCGGACCATCTG
	Fc R2**:** GCA**CGTCTC**AAAGCTCACTTTCCAGGAGAAAGAG
	EPO F1: GCA**GGTCTC**AAGGTGCTCCACCACGTCTTATC
	EPO R4: GCA**GGTCTC**TCTCCATCGCCAGTACGAC
Fc-EPO	Fc-EPO F1: GCA**GGTCTC**AAGGTACTTGTCCACCATGTCC
	Fc-EPO R1: GACGTGGTGGAGCCTTTCCAGGAG
	Fc-EPO F2: CTCCTGGAAAGGCTCCACCACGTC
	Fc-EPO R2: TCG**GGTCTC**AAAGCTCAACGATCGCCAGTACGAC
EPO-mIgG3	mIgG3 F1: CTCGAG**CGTCTC**TAGGTA**GAGACC**GAATTC**GGTCTC**ACCTAGAATACCCAAGCCCAGTACC
	mIgG3 R1: GCGGCCG**CGTCTC**AAAGCTCATTTACCAGGGGAGCGAGACAG
	EPO F1: GCA**GGTCTC**AAGGTGCTCCACCACGTCTTATC
	EPO R5: GCA**GGTCTC**TTAGGACGATCGCCAGTACGAC
	EPO R6: GCA**GGTCTC**TTGTCACGATCGCCAGTACGAC
EPO-hyFc	EPO F1: GCA**GGTCTC**AAGGTGCTCCACCACGTCTTATC
	EPO R7: GCA**GGTCTC**TTCCTACGATCGCCAGTACGAC
EPO-mFc	LFc F1: GAG**CGTCTC**TAGGTA**GAGACC**GGGATCC**GGTCTC**TGGAGGTGGTGGTTCTGGTGGTGGTGGTTCACTTGGCGGACCATCTG
	mFc F1: CCAAAGCCAAGGGTCAACCTAGAGAACCTCAAGTGTACACCCTGCCACCTTC
	mFc R1: GGTTCTCTAGGTTGACCCTTGGCTTTGGAAATAG
	CH3 R1: GCA**CGTCTC**AAAGCCTACTTACCAGGAGACAGG
	EPO F1: GCA**GGTCTC**AAGGTGCTCCACCACGTCTTATC
	EPO R4: GCA**GGTCTC**TCTCCATCGCCAGTACGAC
EPO-CL	CL F1: **GAGCGTCTC**TAGGTA**GAGACC**GGATCC**GGTCTC**TCGTACTGTTGCAGCTC
	CL R1: GCA**GGTCTC**AAAGCTTAGCATTCACCTCGATTAAAG
	EPO F1: GCA**GGTCTC**AAGGTGCTCCACCACGTCTTATC
	EPO R5: GCA**GGTCTC**TTACGACGATCGCCAGTACG

Figure S1: Peptide sequences of the fusion proteins transiently expressed in *N. benthamiana* in the course of this investigation. Green: barley alpha amilase SP; Yellow: EPO domain; Blue: CH2-CH3 domain of Fc; Magenta: CL domain; Grey: Linker. Mutations on CH3 domain are in red.

EPO-^{T}Fc

MANKHLSLSLFLVLLGLSASLASGAPPRLICDSRVLERYLLEAKEAENITTGCAEHCSLNENITVPDTKVNFYAWKRM
EVGQQAVEVWQGLALLSEAVLRGQALLVNSSQPWEPLQLHVDKAVSGLRSLTTLLRALGAQKEAISPPDAASAAPLRT
ITADTFRKLFRVYSNFLRGKLKLYTGEACRTGDRTCPPCPAPELLGGPSVFLFPPKPKDTLMISRTPEVTCVVVDVSH
EDPEVKFNWYVDGVEVHNAKTKPREEQYNSTYRVVSVLTVLHQDWLNGKEYKCKVSNKALPAPIEKTISKAKGQPREP
QVYTLPPSRDELTKNQVSLTCLVKGFYPSDIAVEWESNGQPENNYKTTPPVLDSDGSFFLYSKLTVDKSRWQQGNVFS
CSVMHEALHNHYTQKSLSLSPGK

EPO-^{N}Fc

MANKHLSLSLFLVLLGLSASLASGAPPRLICDSRVLERYLLEAKEAENITTGCAEHCSLNENITVPDTKVNFYAWKRM
EVGQQAVEVWQGLALLSEAVLRGQALLVNSSQPWEPLQLHVDKAVSGLRSLTTLLRALGAQKEAISPPDAASAAPLRT
ITADTFRKLFRVYSNFLRGKLKLYTGEACRTGDRAPELLGGPSVFLFPPKPKDTLMISRTPEVTCVVVDVSHEDPEVK
FNWYVDGVEVHNAKTKPREEQYNSTYRVVSVLTVLHQDWLNGKEYKCKVSNKAFPAPIEKTISKAKGQPREPQVYTLP
PSRDELTKNQVSLTCLVKGFYPSDIAVEWESNGQPENNYKTTPPVLDSDGSFFLYSKLTVDKSRWQQGNVFSCSVMHE
ALHNHYTQKSLSLSPGK

EPO-^{F}Fc

MANKHLSLSLFLVLLGLSASLASGAPPRLICDSRVLERYLLEAKEAENITTGCAEHCSLNENITVPDTKVNFYAWKRM
EVGQQAVEVWQGLALLSEAVLRGQALLVNSSQPWEPLQLHVDKAVSGLRSLTTLLRALGAQKEAISPPDAASAAPLRT
ITADTFRKLFRVYSNFLRGKLKLYTGEACRTGDREPKSCDKTHTCPPCPAPELLGGPSVFLFPPKPKDTLMISRTPEV
TCVVVDVSHEDPEVKFNWYVDGVEVHNAKTKPREEQYNSTYRVVSVLTVLHQDWLNGKEYKCKVSNKALPAPIEKTIS
KAKGQPREPQVYTLPPSRDELTKNQVSLTCLVKGFYPSDIAVEWESNGQPENNYKTTPPVLDSDGSFFLYSKLTVDKS
RWQQGNVFSCSVMHEALHNHYTQKSLSLSPGK

EPO-^{H3}Fc

MANKHLSLSLFLVLLGLSASLASGAPPRLICDSRVLERYLLEAKEAENITTGCAEHCSLNENITVPDTKVNFYAWKRM
EVGQQAVEVWQGLALLSEAVLRGQALLVNSSQPWEPLQLHVDKAVSGLRSLTTLLRALGAQKEAISPPDAASAAPLRT
ITADTFRKLFRVYSNFLRGKLKLYTGEACRTGDEPKSCDTPPPCPRCAPELLGGPSVFLFPPKPKDTLMISRTPEVTC
VVVDVSHEDPEVKFNWYVDGVEVHNAKTKPREEQYNSTYRVVSVLTVLHQDWLNGKEYKCKVSNKAFPAPIEKTISKA
KGQPREPQVYTLPPSRDELTKNQVSLTCLVKGFYPSDIAVEWESNGQPENNYKTTPPVLDSDGSFFLYSKLTVDKSRW
QQGNVFSCSVMHEALHNHYTQKSLSLSPGK

EPO-^{L}Fc

MANKHLSLSLFLVLLGLSASLASGAPPRLICDSRVLERYLLEAKEAENITTGCAEHCSLNENITVPDTKVNFYAWKRM
EVGQQAVEVWQGLALLSEAVLRGQALLVNSSQPWEPLQLHVDKAVSGLRSLTTLLRALGAQKEAISPPDAASAAPLRT
ITADTFRKLFRVYSNFLRGKLKLYTGEACRTGDGGGGSGGGGSLGGPSVFLFPPKPKDTLMISRTPEVTCVVVDVSHE
DPEVKFNWYVDGVEVHNAKTKPREEQYNSTYRVVSVLTVLHQDWLNGKEYKCKVSNKALPAPIEKTISKAKGQPREPQ
VYTLPPSRDELTKNQVSLTCLVKGFYPSDIAVEWESNGQPENNYKTTPPVLDSDGSFFLYSKLTVDKSRWQQGNVFSC
SVMHEALHNHYTQKSLSLSPGK

Fc-EPO

MANKHLSLSLFLVLLGLSASLASGTCPPCPAPELLGGPSVFLFPPKPKDTLMISRTPEVTCVVVDVSHEDPEVKFNWY
VDGVEVHNAKTKPREEQYNSTYRVVSVLTVLHQDWLNGKEYKCKVSNKALPAPIEKTISKAKGQPREPQVYTLPPSRD
ELTKNQVSLTCLVKGFYPSDIAVEWESNGQPENNYKTTPPVLDSDGSFFLYSKLTVDKSRWQQGNVFSCSVMHEALHN
HYTQKSLSLSPGAAPPRLICDSRVLERYLLEAKEAENITTGCAEHCSLNENITVPDTKVNFYAWKRMEVGQQAVEVWQ
GLALLSEAVLRGQALLVNSSQPWEPLQLHVDKAVSGLRSLTTLLRALGAQKEAISPPDAASAAPLRTITADTFRKLFR
VYSNFLRGKLKLYTGEACRTGDR

EPO-mIgG3

MANKHLSLSLFLVLLGLSASLASGAPPRLICDSRVLERYLLEAKEAENITTGCAEHCSLNENITVPDTKVNFYAWKRM
EVGQQAVEVWQGLALLSEAVLRGQALLVNSSQPWEPLQLHVDKAVSGLRSLTTLLRALGAQKEAISPPDAASAAPLRT
ITADTFRKLFRVYSNFLRGKLKLYTGEACRTGDRPRIPKPSTPPGSSCPPGNILGGPSVFIFPPKPKDALMISLTPKV
TCVVVDVSEDDPDVHVSWFVDNKEVHTAWTQPREAQYNSTFRVVSALPIQHQDWMRGKEFKCKVNNKALPAPIERTIS
KPKGRAQTPQVYTIPPPREQMSKKKVSLTCLVTNFFSEAISVEWERNGELEQDYKNTPPILDSDGTYFLYSKLTVDTD
SWLQGEIFTCSVVHEALHNHHTQKNLSRSPGK

EPO-HyFc

MANKHLSLSLFLVLLGLSASLASGAPPRLICDSRVLERYLLEAKEAENITTGCAEHCSLNENITVPDTKVNFYAWKRM
EVGQQAVEVWQGLALLSEAVLRGQALLVNSSQPWEPLQLHVDKAVSGLRSLTTLLRALGAQKEAISPPDAASAAPLRT
ITADTFRKLFRVYSNFLRGKLKLYTGEACRTGDRRNTGRGGEEKKKEKEKEEQEERETKTPECP**SHTQPLGV**FLFPPK
PKDTLMISRTPEVTCVVVDVSQEDPEVQFNWYVDGVEVHNAKTKPREEQFNSTYRVVSVLTVLHQDWLNGKEYKCKVS
NKGLPSSIEKTISKAKGQPREPQVYTLPPSQEEMTKNQVSLTCLVKGFYPSDIAVEWESNGQPENNYKTTPPVLDSDG
SFFLYSRLTVDKSRWQEGNVFSCSVMHEALHNHYTQKSLSLSLGK

EPO-mFc

MANKHLSLSLFLVLLGLSASLASGAPPRLICDSRVLERYLLEAKEAENITTGCAEHCSLNENITVPDTKVNFYAWKRM
EVGQQAVEVWQGLALLSEAVLRGQALLVNSSQPWEPLQLHVDKAVSGLRSLTTLLRALGAQKEAISPPDAASAAPLRT
ITADTFRKLFRVYSNFLRGKLKLYTGEACRTGDGGGGSGGGGSLGGPSVFLFPPKPKDTLMISRTPEVTCVVVDVSHE
DPEVKFNWYVDGVEVHNAKTKPREEQY**N**STYRVVSVLTVLHQDWLNGKEYKCKVSNKALPAPIEKTISKAKGQPREPQ
VYTLPPSRDELTKNQVSL**R**C**H**VKGFYPSDIAVEWESNGQPENNYKTT**K**PVLDSDGSF**R**LYSKLTVDKSRWQQGNVFSC
SVMHEALHNHYTQKSLSLSPGK-

EPO-CL

MANKHLSLSLFLVLLGLSASLASGAPPRLICDSRVLERYLLEAKEAENITTGCAEHCSLNENITVPDTKVNFYAWKRM
EVGQQAVEVWQGLALLSEAVLRGQALLVNSSQPWEPLQLHVDKAVSGLRSLTTLLRALGAQKEAISPPDAASAAPLRT
ITADTFRKLFRVYSNFLRGKLKLYTGEACRTGDRRTVAAPSVFIFPPSDEQLKSGTASVVCLLNNFYPREAKVQWKVD
NALQSGNSQESVTEQDSKDSTYSLSSTLTLSKADYEKHKVYACEVTHQGLSSPVTKSFNRGEC

ScFvFc

EVQLVESGGGLIQPGGSLRLSCVASGFRFSSHEMNWVRQAPGKGLEWVSYIGSRGSDTSYADSVKGRFTVSRDNARNT
LYLQMNNLRAEDTAVYYCARERYRYFEDYYHGLDVWGQGTTVTVSSGSSGGGGSGGGGSGGSALETTLTQSPGTLSLS
PGERATLSCRASQSVSSSYLAWYQQKPGQAPRLLIYGASSRATGIPDRFSGSGSGTDFTLTISRLEPEDFAVYYCQQY
GSSPLTFGGGTKVEIKRAAAGTGSGSAEPKSSDKTHTCPPCPAPELLGGPSVFLFPPKPKDTLMISRTPEVTCVVVDV
SHEDPEVKFNWYVDGVEVHNAKTKPREEQYNSTYRVVSVLTVLHQDWLNGKEYKCKVSNKALPAPIEKTISKAKGQPR
EPQVYTLPPSREEMTKNQVSLTCLVKGFYPSDIAVEWESNGQPENNYKTTPPVLDSDGSFFLYSKLTLDKSRWQQGNV
FSCSVMHEALHNHYTQKSLSLSPGK

Rx-Hc

MGWSLILLFLVAVATRVLSQVQLQQPGAELVKPGASVKMSCKASGYTFTSYNMHWVKQTPGRGLEWIGAIYPGNGDTS
YNQKFKGKATLTADKSSSTAYMQLSSLTSEDSAVYYCARSTYYGGDWYFNVWGAGTTVTVSAASTKGPSVFPLAPSSK
STSGGTAALGCLVKDYFPEPVTVSWNSGALTSGVHTFPAVLQSSGLYSLSSVVTVPSSSLGTQTYICNVNHKPSNTKV
DKKAEPKSCDKTHTCPPCPAPELLGGPSVFLFPPKPKDTLMISRTPEVTCVVVDVSHEDPEVKFNWYVDGVEVHNAKT
KPREEQYNSTYRVVSVLTVLHQDWLNGKEYKCKVSNKALPAPIEKTISKAKGQPREPQVYTLPPSRDELTKNQVSLTC
LVKGFYPSDIAVEWESNGQPENNYKTTPPVLDSDGSFFLYSKLTVDKSRWQQGNVFSCSVMHEALHNHYTQKSLSLSP
GK

Figure S2: Analysis of the expression EPO-IgG1 variants in the apoplast.
Intracellular fluids (apoplast, AF) from *N. benthamiana* ΔXTFT leaves expressing different EPO-Fc variants analysed by immunoblotting at 5 days post infiltration (dpi) using anti-EPO antibodies. Protein size markers are shown in kilo Dalton (kDa).

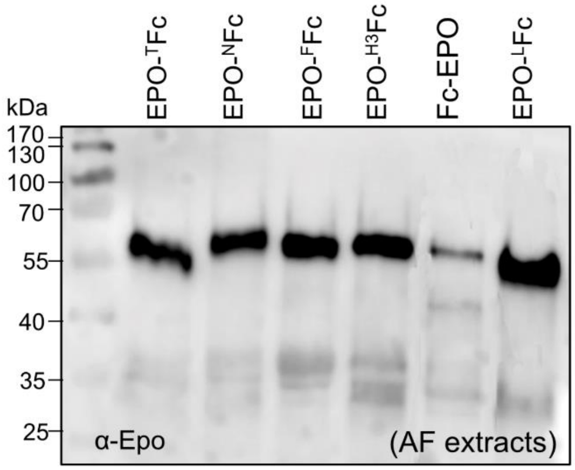

Figure S3: Analysis of the expression ^{L}Fc (non-fused). Total soluble proteins extracts (TSP) from *N. benthamiana* ΔXTFT leaves expressing ^{L}Fc (non-fused) were analysed in 8% SDS-PAGE under reducing (R) and non-reducing (NR) conditions and in native 12% PAGE with anti-hIgG antibodies. Protein size markers are shown in kilo Dalton (kDa).

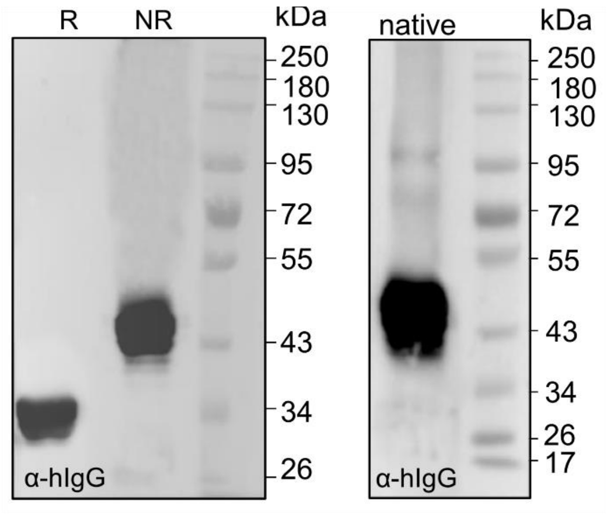

Figure S4: Site-specific *N*-glycosylation profiles.
Mass spectra of tryptic glycopeptides of EPO-^{L}Fc, EPO-mFc and EPO-CL expressed in *N. benthamiana* ΔXT/FT. N-glycosylation profiles of the EPO glycopeptide 2 (QALLVNSSQPWEPLQLHVDK); and Fc glycopeptide (EQYNSTYR) are shown. The major glycosylated peaks are depicted. Symbol nomenclature in accordance with the Consortium of Functional Glycomics (http://glycomics.scripps.edu/CFGnomenclature.pdf). Illustrations display the main glycoforms.

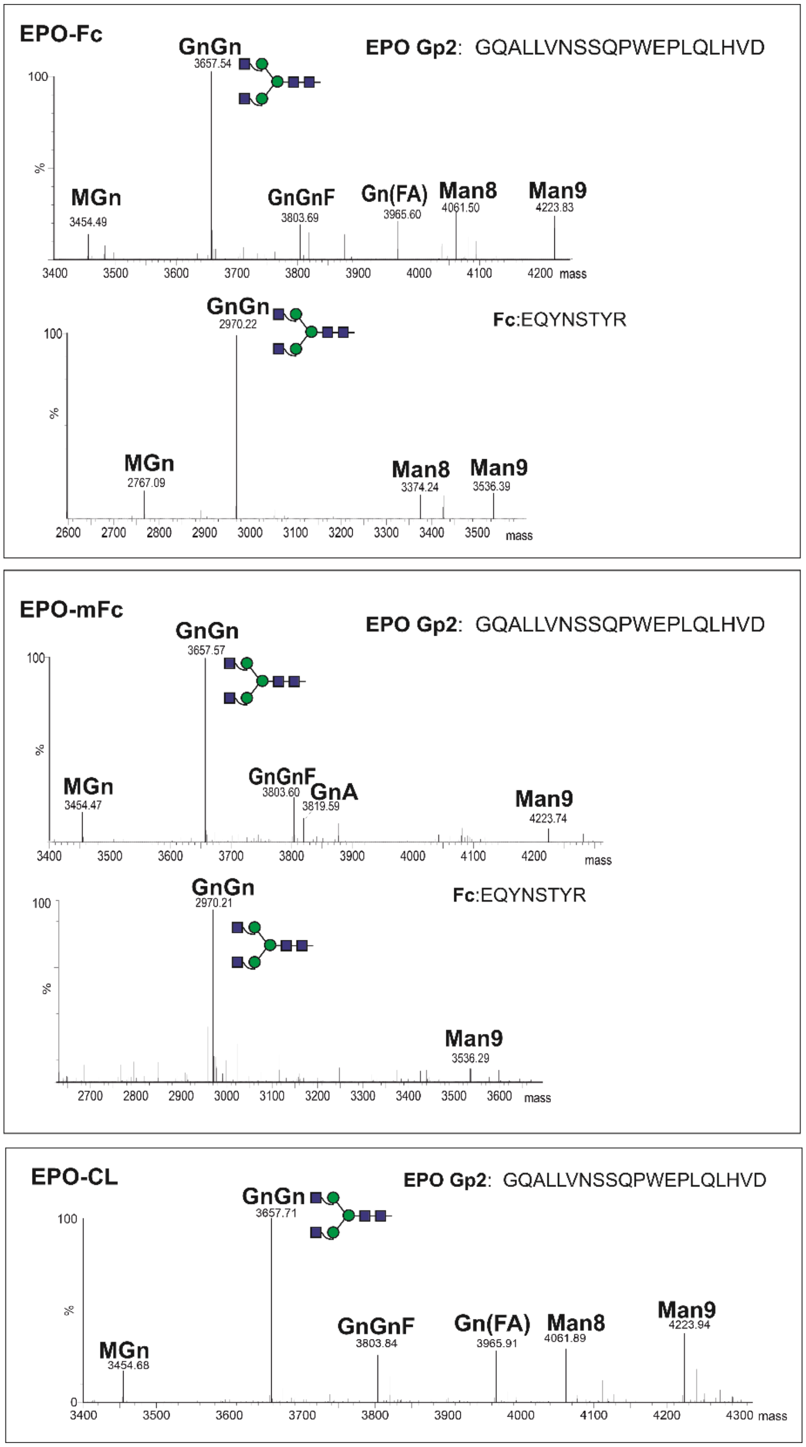

Supplement: Supplementary Figure 1 — Peptide sequence of the proteins analyzed in this investigation. [file Data_Sheet_1.PDF]
